# Inhibition of EGFR-AKT Axis Results in the Suppression of Ovarian Tumors *In Vitro* and in Preclinical Mouse Model

**DOI:** 10.1371/journal.pone.0043577

**Published:** 2012-08-27

**Authors:** Sivakumar Loganathan, Prabodh K. Kandala, Parul Gupta, Sanjay K. Srivastava

**Affiliations:** 1 Department of Pharmacology and University of Pittsburgh Cancer Institute, University of Pittsburgh, Pennsylvania, United States of America; 2 Department of Biomedical Sciences and Cancer Biology Center, Texas Tech University Health Sciences Center, Amarillo, Texas, United States of America; Bauer Research Foundation, United States of America

## Abstract

Ovarian cancer is the leading cause of cancer related deaths in women. Genetic alterations including overexpression of EGFR play a crucial role in ovarian carcinogenesis. Here we evaluated the effect of phenethyl isothiocyanate (PEITC) in ovarian tumor cells *in vitro* and *in vivo*. Oral administration of 12 µmol PEITC resulted in drastically suppressing ovarian tumor growth in a preclinical mouse model. Our *in vitro* studies demonstrated that PEITC suppress the growth of SKOV-3, OVCAR-3 and TOV-21G human ovarian cancer cells by inducing apoptosis in a concentration-dependent manner. Growth inhibitory effects of PEITC were mediated by inhibition of EGFR and AKT, which are known to be overexpressed in ovarian tumors. PEITC treatment caused significant down regulation of constitutive protein levels as well as phosphorylation of EGFR at Tyr1068 in various ovarian cancer cells. In addition, PEITC treatment drastically reduced the phosphorylation of AKT which is downstream to EGFR and disrupted mTOR signaling. PEITC treatment also inhibited the kinase activity of AKT as observed by the down regulation of p-GSK in OVCAR-3 and TOV-21G cells. AKT overexpression or TGF treatment blocked PEITC induced apoptosis in ovarian cancer cells. These results suggest that PEITC targets EGFR/AKT pathway in our model. In conclusion, our study suggests that PEITC could be used alone or in combination with other therapeutic agents to treat ovarian cancer.

## Introduction

Ovarian cancer is one of the leading causes of gynecologic cancer-related deaths among women in western countries [1]. The cause of ovarian cancer is not clear and it is often detected at an advanced stage. The overall prognosis of ovarian cancer is very poor despite significant advances in surgical and therapeutic management [2]. The current standard of care includes cytoreduction followed by cytotoxic chemotherapy. However, recurrence remains a significant problem [3]. The most common form of ovarian cancer arises from ovarian surface epithelium. Epidermal growth factor receptor (EGFR) is commonly expressed in ovarian surface epithelium [4].

Activation of various tyrosine kinases including EGFR is important in ovarian cancer pathogenesis. Approximately 70% of ovarian tumors express activated EGFR [5]. EGFR is a trans-membrane receptor whose activation is a highly conserved process. Various ligands such as EGF and TGF activate EGFR. EGFR plays a significant role in neural development and formation of skin. However, in cancer cells, EGFR is involved in various pro-survival and anti-apoptotic pathways [6]–[8]. Furthermore, EGFR is also involved in cell migration, metastasis, angiogenesis and EMT [9]–[11].

One of the major downstream pathways that are regulated by EGFR is AKT. Activation of EGFR leads to the activation of AKT by its phosphorylation at Ser-473 [12], [13]. AKT is frequently activated or overexpressed in ovarian tumors [14], [15] and plays a major role in ovarian carcinogenesis. The overexpression of AKT is frequently associated with poor prognosis and more aggressive phenotype. Like EGFR, AKT also plays a major role in angiogenesis, metastasis and anti-apoptosis. Since EGFR and AKT are involved in various aspects of cancer growth ranging from tumor initiation, angiogenesis, and metastasis, EGFR-AKT axis represents an attractive target for therapeutic intervention.

PEITC is a major isothiocyanate present in cruciferous vegetables [16]. Accumulating epidemiological evidence indicates an inverse relationship between intake of cruciferous vegetables and the risk of ovarian cancer [17], [18]. Several studies, including those from our laboratory suggested that various isothiocyanates possess chemo-preventive and therapeutic properties [16], [19], [20]. PEITC in particular was shown to be effective against prostate, cervical and lung cancers [21]–[23]. Interestingly, PEITC is in clinical trials for lung cancer. In the present study, we investigated the mechanism by which PEITC inhibits the proliferation of ovarian cancer cells and evaluated its efficacy *in vivo* in a tumor xenograft model.

**Figure 1:**
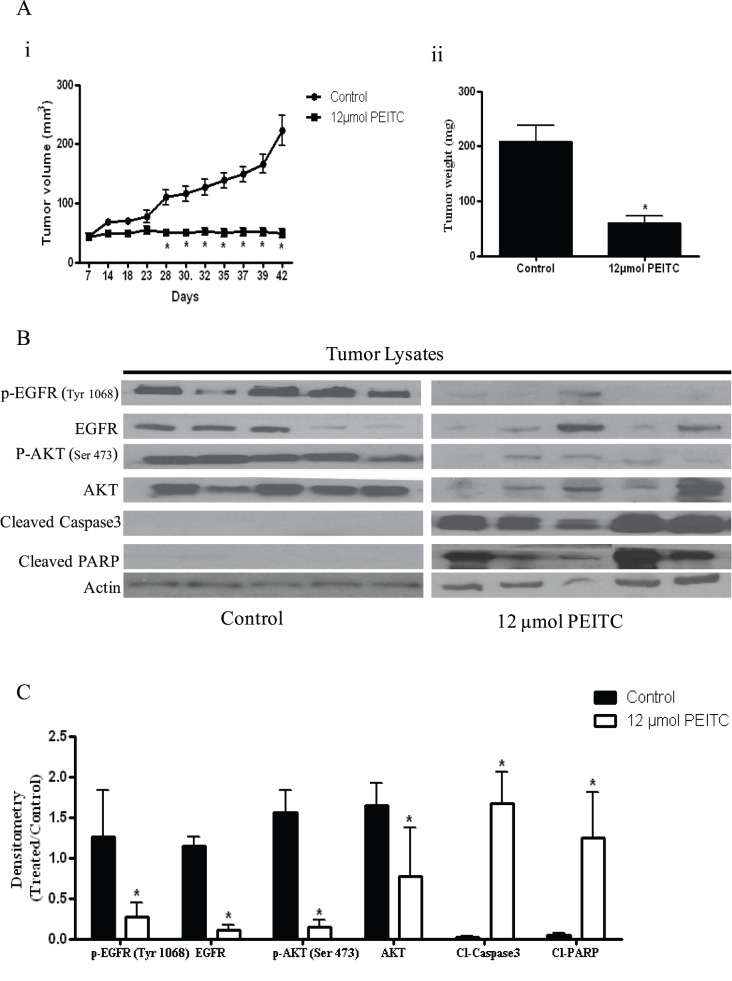
PEITC suppresses the growth of ovarian tumor xenografts by inhibiting EGFR-AKT pathway. SKOV-3 tumor cells were implanted into athymic nude mice and randomized into two groups. Mice received PBS or 12 µmol PEITC by oral gavage every day until day 42. (A–i) Effect of PEITC on tumor growth. (A-ii) Tumor weight from control and treatment groups (B) Inhibition of EGFR signaling in the tumors of mice administered with PEITC. Tumors from control and treated mice were excised at day 42, lysed and analyzed by western blotting for p-EGFR (Tyr-1068), EGFR, p-AKT (Ser-473), AKT, Cl-Caspase 3 and Cl-PARP. Blots were stripped and reprobed with actin antibody to verify equal protein loading. Each lane represents a different tumor sample. (C) Densitometric quantitation of western blotting represented above. The differences between the groups were compared by student’s t-test. Statistical tests were two sided. *p<0.05 when compared to control.

## Materials and Methods

### Chemicals

Antibodies against Cl-caspase3, Cl-PARP, p-EGFR (Tyr-1068), EGFR, p-AKT (Ser-473), p- mTOR (Ser 2481), Raptor and AKT antibodies were obtained from Cell Signaling Technology (Danvers, MA). Rictor antibody was obtained from Novusbio (Littleton, CO). PEITC, actin antibody, TGF, SRB, MCDB105 and Medium 199 were procured from Sigma Aldrich (St. Louis, MO). RPMI and McCoy 5A were purchased from Mediatech (Manassas, VA). AnnexinV apoptosis kit was procured from BD biosciences (San Jose, CA).

**Figure 2:**
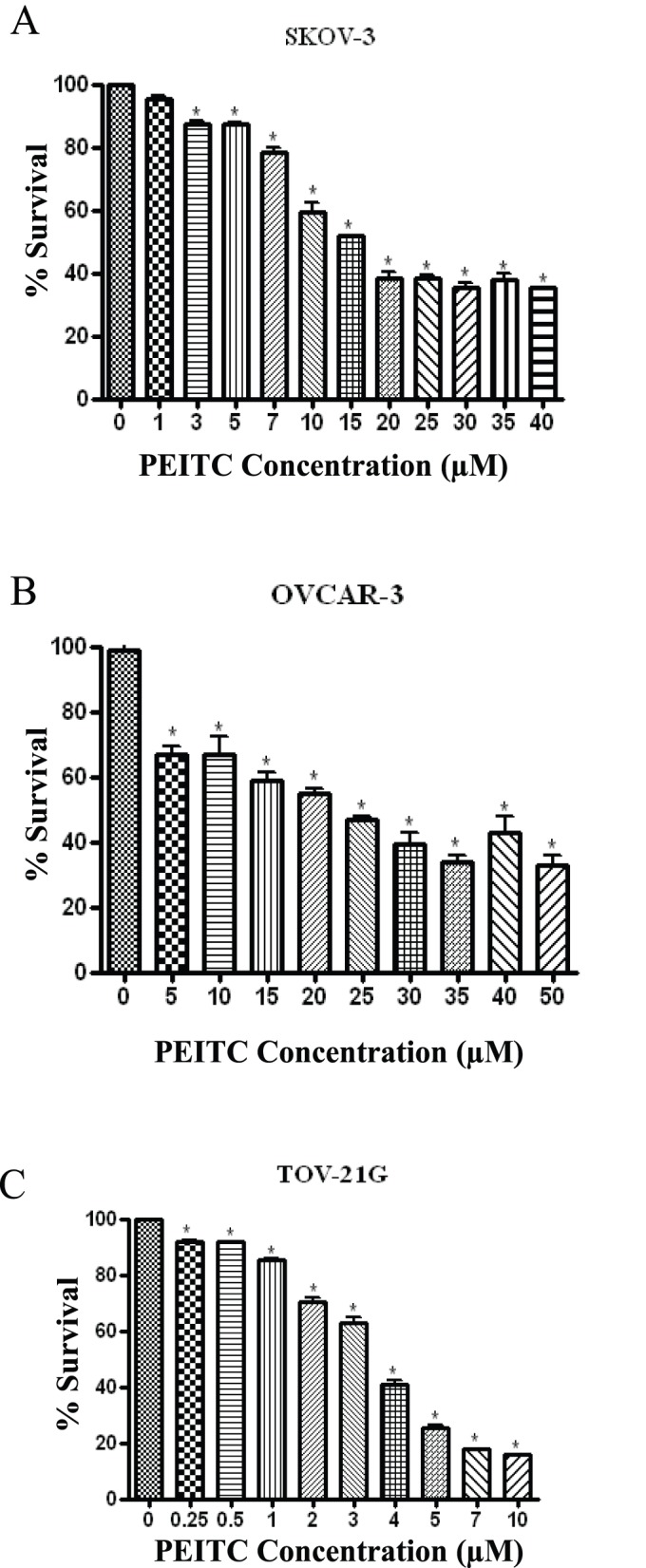
PEITC is cytotoxic to human ovarian cancer cells. Effect of varying concentrations of PEITC for 24 hours on (A) SKOV-3, (B) OVCAR-3 and (C) TOV-21G cells was determined by Sulphorhodamine B cell survival assay. Values are means ± SEM of 2 independent experiments with 8 replicates. *p<0.05 when compared to control.

### *In vivo* Tumor Xenograft

Four to six week old female athymic nude mice were purchased from Charles River Laboratories (Wilmington, MA). Institutional Animal Care and Use Committee (IACUC), Texas Tech University Health Sciences Center approved the use of mice and their treatment, and all the experiments were carried out in strict compliance with regulations. Mice were fed with antioxidant-free AIN-76A special diet for a week before starting the experiment. About 5×10^6^ SKOV-3 cells were injected subcutaneously into both right and left flanks. Ten mice were assigned randomly to each group. Since each mouse was implanted two xenografts, each group had twenty tumors. Mice in the control group received PBS, whereas, mice in the treatment group received 12 µmol PEITC suspended in PBS by oral gavage every day. Tumor growth was monitored until day 42 as described by us previously [16], [24]. At day 42, mice were euthanized and tumors were removed, weighed and processed for western blot analysis.

### Cell Cultures

SKOV-3, OVCAR-3, TOV-21G cells lines were procured from American Type Culture Collection (ATCC, Manassas, VA). SKOV-3 cells were maintained in McCoy’s 5A medium supplemented with 10% Fetal Bovine Serum (FBS). OVCAR-3 cells were maintained in RPMI medium supplemented with 20% FBS, 10 mM sodium pyruvate, 10 mM HEPES, 10 mg/L bovine insulin and 4.5 g/L glucose. TOV21G cells were maintained in 1∶1 mixture of MCDB105 and Medium 199 supplemented with 15% FBS. A 1% antibiotic mixture was used in all the above media. All the cell lines were maintained at 37°C in a humidified incubator circulated with 5% CO_2_/95% air.

**Figure 3:**
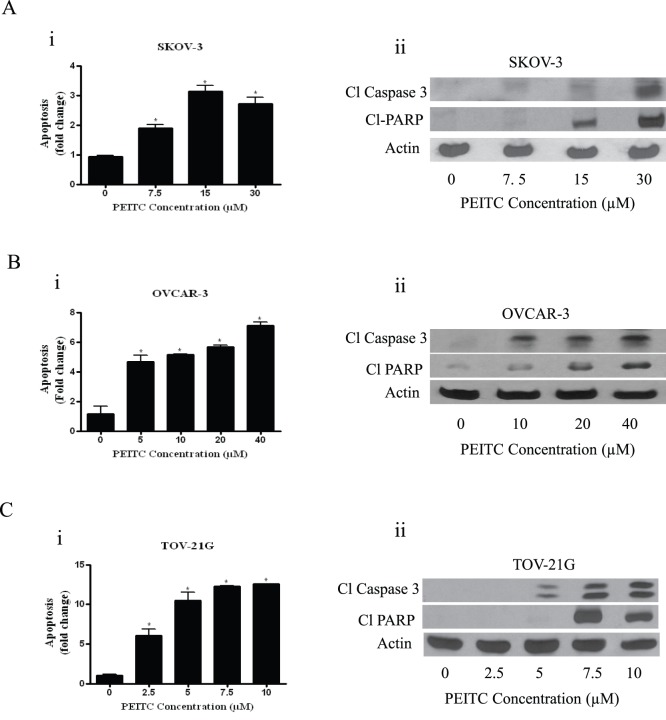
PEITC induces apoptosis in ovarian cancer cells. SKOV-3, OVCAR-3, TOV-21G, cells were treated with varying concentrations of PEITC for 24 hours. Flowcytometry and western blotting were used to determine apoptosis. (A) SKOV-3, (B) OVCAR-3 and (C) TOV-21G cells treated with varying concentrations of PEITC.

### Sulphorhodamine B Cell Survival Assay

About 5,000 cells in 0.1 ml medium were plated in 96 well plates and allowed to attach overnight. Desired concentrations of PEITC were added to the cells and incubated at 37°C for 24 h. The cells were then processed and stained with 0.4% SRB solution and the absorbance was read at 570 nm using a Biotek plate reader as described by us previously [25].

### Western Blotting

SKOV-3, OVCAR-3 and TOV-21G cells were exposed to varying concentrations of PEITC alone or in presence of TGF. In another experiment, cells were treated with 15 µM PEITC for different time intervals. For concentration dependent study SKOV3 cells were treated with 7.5, 15 and 30 µM PEITC for 24 hours. Cells were collected, lysed, and 20–80 µg of protein was subjected to SDS gel electrophoresis followed by immunoblotting as described by us previously [20], [25].

**Figure 4:**
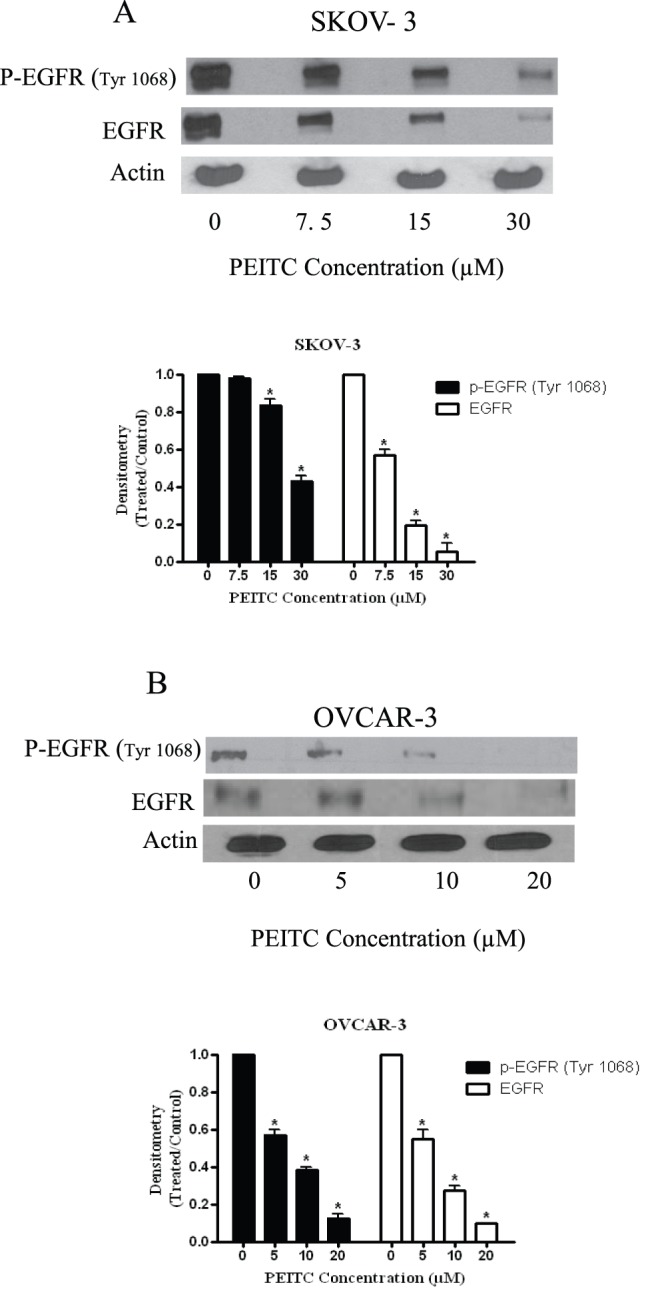
PEITC inhibits the activation of EGFR in ovarian cancer cells. Representative blots showing the concentration dependent effect of PEITC on p-EGFR (Tyr-1068) and EGFR in (A) SKOV-3 and (B) OVCAR-3 ovarian cancer cells. Actin was used as loading control. Each *p<0.05 when compared to control.

### Annexin-V Apoptosis Assay

SKOV-3, OVCAR-3 or TOV-21G cells were plated at a density of 0.3×10^6^ cells per well in a six-well plate and allowed to attach overnight. Cells were then treated with or without PEITC. After 24 hours, cells were washed, suspended in binding buffer and incubated for 15 minutes with Annexin V-FITC. Fluorescence was measured using C6 Accuri flow cytometer (Ann Arbor, MI) with a minimum of 10,000 events per sample as described by us previously [16], [26].

### Immunoprecipitation Assay

Immunoprecipitation assay was performed to examine the effect of PEITC on the complex of mTOR with Rictor and Raptor. Briefly, SKOV3 cells were treated with 15 µM PEITC for 24 hours. The cell lysates from control and PEITC treated cells were immunoprecipitated with the mTOR antibody (Cell Signaling), as described by us earlier [27]. The proteins were separated by SDS-polyacrylamide gel and transferred to PVDF membrane. The membranes were immnobloted for Rictor, Raptor and mTOR using respective antibodies.

**Figure 5:**
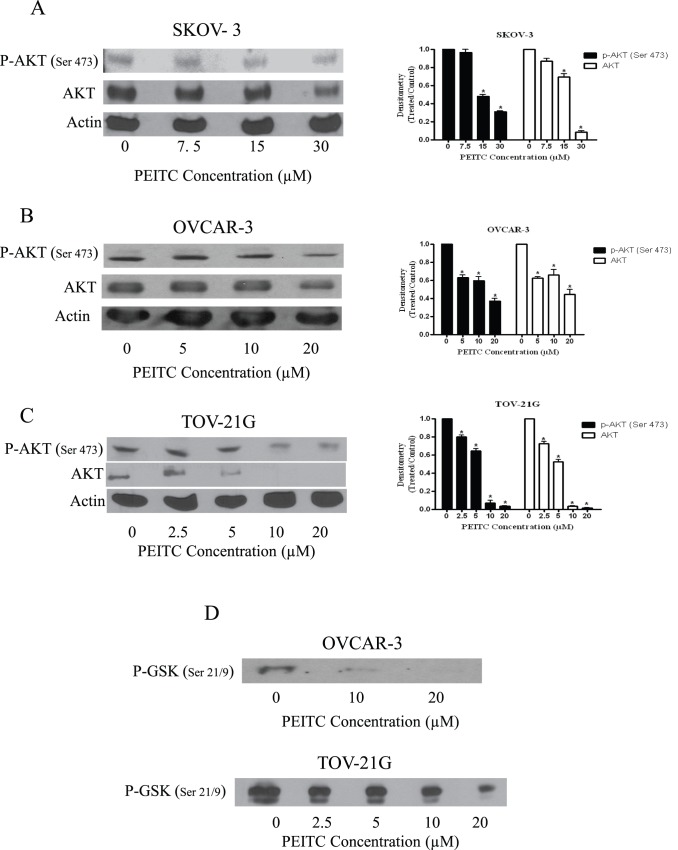
PEITC inhibits AKT in ovarian cancer cells. Representative blots of the concentration dependent effect of PEITC on p-AKT (Ser-473) and AKT in (A) SKOV-3, (B) OVCAR-3 and (C) TOV-21G ovarian cancer cells. Actin was used as loading control. *p<0.05 when compared to control. (D) Concentration dependent effect of PEITC for 24 h on AKT kinase activity as represented by phosphorylation of GSK at Ser-21/9 in OVCAR-3 or TOV-21G cells.

### TGF Treatment

SKOV-3 or OVCAR-3 cells were treated with 50 ng/ml TGF for 1 hour followed by incubation with different concentrations of PEITC for 24 hours. Cells were then processed for western blotting as described above.

### AKT Overexpression

SKOV-3 cells were transiently transfected with plasmid containing wil-type AKT (a generous gift from Daniel Altschuler, University of Pittsburgh, Pittsburgh, Pennsylvania) by using Fugene (Roche). Briefly, 0.3×10^5^ cells were transfected with 0.5 µg of the AKT plasmid diluted in Opti-MEM serum-free medium to which Fugene reagent was added before the mixture was added to cells. Cells were incubated with plasmid-Fugene mixture for 6 hours and then media was replaced with fresh growth medium and incubated for another 24 hours. Transfected cells were treated with 10 µM PEITC for 24 hours. Whole cell lysates were subjected to Western blot analysis and actin was used as loading control.

**Figure 6:**
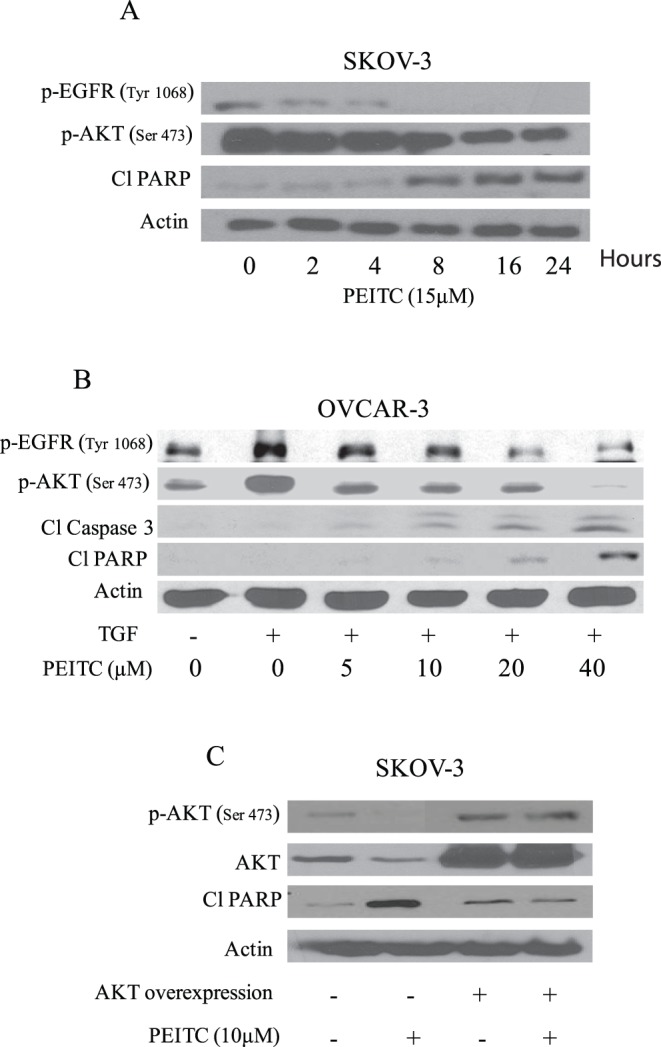
TGF treatment of AKT overexpression overrides the effects of PEITC. (A) Representative blot of time dependent effect of 15 µM PEITC on p-EGFR (Tyr 1068), p-AKT (Ser-473), Cl-PARP in SKOV-3 ovarian cancer cells. Actin was used as loading control. (B) OVCAR-3 cells were stimulated with 50 ng/mL TGF for 1 hour after treatment with varying concentrations of PEITC for 24 hours. Whole-cell lysates were resolved on 10% SDS-PAGE for the analysis of phosphorylation of EGFR at Tyr-1068, phosphorylation of AKT at Ser-473, and cleavage of caspase-3 and PARP. Actin was used as a control for loading. Effect of AKT overexpression on PEITC induced apoptosis was also determined in SKOV-3 cells. (C) Representative blots showing p-AKT (Ser 473), AKT and Cl-PARP. Actin was used as a loading control.

### Quantitation and Statistical Analysis

All the statistical analysis was performed using Prism 5.0 (GraphPad Software Inc., San Diego, CA). The data represents mean ± S.D. Student’s *t*-test was used to compare the control and treated groups. In experiments involving more than three groups, non-parametric analysis of variance followed by Bonferroni post hoc multiple comparison test was used. All statistical tests were two sided. Differences were considered statistically significant when the p value was less than 0.05.

**Figure 7:**
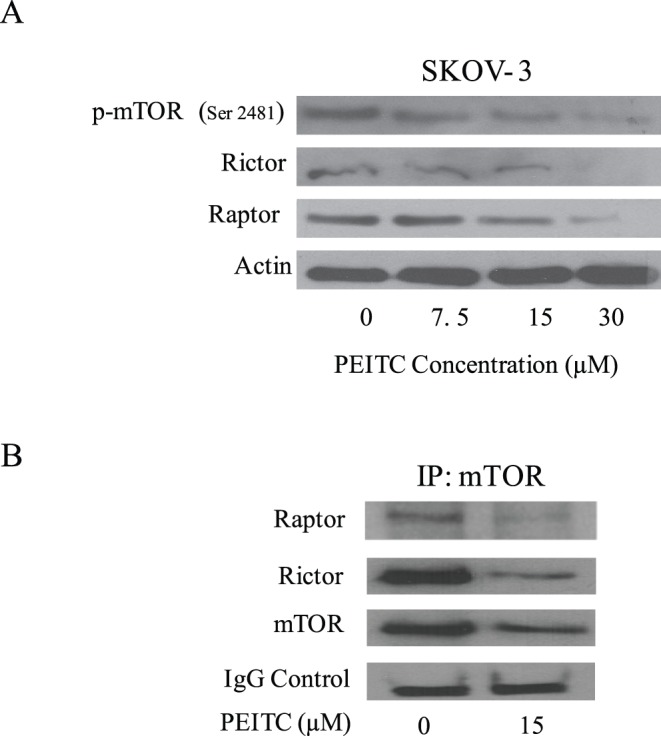
PEITC treatment inhibits mTOR, Raptor and Rictor of mTORC1 and mTORC2 complexes. (A) Representative blots of the concentration dependent effect of PEITC (0–7.5 µM) on p-mTOR (Ser-2481), Rictor and Raptor on SKOV-3 ovarian cancer cells. Actin was used as loading control. (B) SKOV3 cells treated with 5 µM PEITC for 24 h were immunoprecipitated with mTOR antibody. The blots were probed with antibodies for Rictor and Raptor.

## Results

### Phenethyl Isothiocyanate (PEITC) Suppresses Ovarian Tumor Growth in a Xenograft Model

Isothiocyanates were shown to be therapeutically active against various malignancies. To test the possibility that PEITC treatment would suppress the growth of ovarian tumors, SKOV-3 tumor xenografts were established in female athymic nude mice by subcutaneously injecting 5×10^6^ cells in both right and left flanks. Mice were randomly divided into control and treatment group. Tumor bearing mice were fed 12 µmol PEITC every day and tumor growth was recorded periodically. Our results demonstrate that PEITC substantially suppressed the growth of ovarian tumors in athymic nude mice. For example, at day 42, the average tumor volume in mice from treatment group was approximately 60 mm^3^, whereas average tumor volume in mice from control group was approximately 220 mm^3^ (Fig. 1A). Both tumor volume and tumor weight in the treatment group was reduced by almost 70% as compared with control groups (Fig. 1A).

### Tumor Growth Inhibition by PEITC was Associated with Blockade of EGFR-AKT Pathway

EGFR-AKT pathway is constitutively activated in majority of ovarian tumors. Over-expression of EGFR or its mutations are reported in approximately 70% of ovarian tumors. Since we observed suppression of ovarian tumors by oral administration of PEITC, we hypothesized that growth inhibitory effects of PEITC in ovarian tumors in vivo were through inhibition of EGFR-AKT. To test our hypothesis, phosphorylation levels of EGFR and AKT were examined in tumor lysates by western blotting. As shown in Figure 1B, phosphorylation of EGFR at Tyr-1068 and AKT at Ser-473 were drastically suppressed by PEITC treatment. Next, we looked for apoptosis in the tumors from PEITC treated mice. As expected, we observed cleaved products of caspase-3 and PARP in the tumors from PEITC treated mice indicating that PEITC induces apoptosis in ovarian tumors *in vivo* (Figure 1B). These observations indicate that tumor growth suppression by PEITC was associated with the inhibition of EGFR-AKT pathway and induction of apoptosis.

### PEITC Inhibits the Proliferation of Ovarian Cancer Cells

To determine the mechanism of PEITC, three different ovarian cancer cells were used. SKOV-3, OVCAR-3 and TOV-21G cells were exposed to varying concentrations of PEITC for 24 h. We observed that PEITC treatment reduced the proliferation of ovarian cancer cells in a concentration dependent manner (Figure 2 A–C). The IC_50_ of PEITC was approximately 15 µM in SKOV-3 cells (Figure 2A). In OVCAR-3 and TOV-21G cells, IC_50_ was observed to be approximately 20 µM and 5 µM respectively (Figure 2 B–C).

### PEITC Induces Apoptosis in Ovarian Cancer Cells

In another experiment, we tested whether growth inhibitory effects of PEITC were concurrent with its apoptosis inducing ability. Treatment of SKOV-3 cells with various concentrations of PEITC resulted in approximately 2 to 5 fold apoptosis over control (Fig. 3A i). To rule out the cell specific effects of PEITC, we evaluated PEITC-induced apoptosis in two other ovarian cancer cell lines. Our results showed that apoptosis induced by PEITC was nearly 4 to 6 fold in OVCAR-3 and 2 to 10 fold in TOV-21G cells (Fig. 3 B–C). These observations clearly establish that PEITC reduces the proliferation of ovarian cancer cells by inducing apoptosis.

Apoptosis inducing effects of PEITC were further confirmed by western blot analysis that revealed cleavage of Caspase-3 and PARP in PEITC- treated ovarian cancer cells (Figure 3 A–C).

### PEITC Inhibits the Activation of EGFR in Ovarian Cancer Cells

To extend the molecular observations made *in vivo*, we treated SKOV-3 and OVCAR-3 cells with PEITC for 24 hours. As shown in Figure 4, PEITC treatment significantly blocked the phosphorylation of EGFR at Tyr-1068 in a concentration dependent manner in all the three cell lines tested. Approximately, 50% decrease in the phosphorylation of EGFR at Tyr-1068 was observed in SKOV-3 cells when treated with 30 µM PEITC (Figure 4A), whereas 65% inhibition was observed in OVCAR-3 cells exposed to 10 µM PEITC for 24 hours (Figure 4B). Interestingly, our results also revealed a dramatic reduction in the protein expression of EGFR in ovarian cancer cells treated with PEITC (Figure 4 A–B). Taken together, these results demonstrate that PEITC blocks the phosphorylation as well as reduce protein levels of EGFR in ovarian cancer cells.

### PEITC Treatment Blocks AKT Activation

EGFR regulates various cellular processes by directly acting on downstream molecules such as AKT. Activation of EGFR leads to the phosphorylation of Akt at Ser 473. Because we observed a significant blockade in EGFR activation by PEITC treatment, we sought to determine the effect of PEITC on both activation and constitutive expression of AKT. Exposure of SKOV-3, OVCAR-3 or TOV-21G cells to different concentrations of PEITC for 24 h resulted in the significant inhibition of the phosphorylation as well as constitutive expression of AKT (Fig. 5 A–C). More than 50% reduction was observed in the phosphorylation of AKT at Ser 473 in SKOV-3 cells (Figure 5A). AKT phosphorylation was reduced by70% and 90% in OVCAR-3 and TOV-21G cells respectively (Figure 5 B–C). PEITC had similar effects on constitutive expression of AKT in all the three ovarian cancer cell lines. These results indicate that PEITC modulates the downstream molecules of EGFR pathway.

### AKT Kinase Activity

Since substantially reduced phosphorylation of AKT was observed by PEITC treatment; we wanted to confirm these observations by AKT kinase activity, which measures the functionality of AKT. AKT activity was determined by evaluating the phosphorylation of its downstream substrate GSK. As shown in Figure 5D, a significant reduction in the kinase activity of AKT was observed in OVCAR-3 and TOV-21G cells. For example, treatment of OVCAR-3 cells with 10 µM PEITC for 24 hours resulted in the inhibition of approximately 80% AKT kinase activity as compared with control cells (Figure 5D).

### Early Inhibition of EGFR by PEITC

To evaluate whether induction of apoptosis by PEITC was associated with EGFR inhibition, SKOV-3 cells were treated with 15 µM PEITC at different time intervals. The reduction in the phosphorylation of EGFR and AKT was observed just after 2 hours of PEITC treatment and this effect increased at later time points (Figure 6A). However, cleavage of PARP was observed only after 8 hours PEITC treatment suggesting that inhibition of EGFR/AKT lead to apoptosis in our model (Figure 6A).

### Activation of EGFR-AKT by TGF is Inhibited by PEITC

EGFR can be activated by growth factors and ligands such as TGF and EGF. When TGF binds, EGFR is phosphorylated which in-turn phosphorylates its downstream molecules such as AKT. Treatment OVCAR-3 cells with 50 ng/ml TGF resulted in the 3 fold activation (phosphorylation) of EGFR and AKT (Fig. 6B). However, PEITC treatment was able to reduce the activation of EGFR and AKT induced by TGF (Figure 6B). The effect of TGF and PEITC on cleavage of caspase-3 and PARP was also evaluated. From Figure 6B, it is clear that PEITC treatment was able to induce cleavage of caspase-3 and PARP in OVCAR-3 cells.

### AKT Overexpression Abrogates the Effects of PEITC

To further strengthen our observation that PEITC-induced apoptosis was mediated by AKT inhibition in ovarian cancer cells, we transiently transfected SKOV-3 cells with AKT encoding plasmid for 24 hours, resulting in almost 7 fold increase in the expression of AKT. Correspondingly, phosphorylation of AKT at Ser-473 also increased substantially. AKT overexpression completely prevented PEITC induced suppression of AKT phosphorylation. Furthermore, AKT overexpression abrogated PEITC induced apoptosis as evaluated by cleavage of PARP (Figure 6C). Taken together, our results clearly indicate that apoptosis induced by PEITC was almost completely blocked in the cells overexpressing AKT, indicating AKT as a target of PEITC in ovarian cancer cells.

### PEITC Inhibits mTORC1 and mTORC2 Complexes

Recent literature suggests that EGFR-AKT activation regulates mTOR activity which is important for cell proliferation 28,29. mTOR is regulated by two complexes- mTORC1 and mTORC2. The mTORC1complex consists of regulatory associated protein of mTOR (Raptor), which binds to mTOR and facilitates mTOR signaling in translation and cell growth [30], [31]. On the other hand, mTORC2 complex consists of Rapamycin insensitive companion of mTOR (Rictor) bound to mTOR. The Rictor-mTOR (mTORC2) complex regulates the phosphorylation of AKT at Ser 473 [32]. Since, we observed that PEITC suppressed the phosphorylation of AKT, we hypothesized PEITC treatment would disturb mTOR signaling. As expected, our results demonstrated that PEITC treatment disrupted mTOR signaling by down-regulating p- mTOR (Ser 2481) and expression of Raptor and Rictor, which are involved in mTORC1 and mTORC2 complexes (Fig. 7A). To confirm the dissociation of the complex, mTOR was immunoprecipitated from control and PEITC treated cells and immunoblotted for Rictor and Raptor. Our results clearly showed that mTOR complex partners Rictor and Raptor were suppressed by PEITC treatment in SKOV3 cells (Fig. 7B). The expression of mTOR-associated Rictor was reduced more than Raptor by PEITC treatment (Fig. 7B). These observations suggest that the mTORC2 complex was affected more by PEITC treatment than mTORC1 complex. Nonetheless, further studies are required to delineate the exact mechanism by which PEITC disrupts mTORC1 and mTORC2 complexes.

## Discussion

In the present study, we showed the mechanism by which PEITC suppressed the growth of ovarian cancer cells in culture as well as in animal model. Our results show that oral administration of 12 µmol PEITC to athymic nude mice significantly suppressed the growth of SKOV-3 ovarian tumor xenografts. Tumor growth suppression by PEITC treatment was associated with increased apoptosis in the tumor cells, which in turn was linked with the inhibition of EGFR and AKT activation. Inhibition of phosphorylation of EGFR and AKT were confirmed in SKOV-3 and OVCAR-3 ovarian cancer cells by western blotting. PEITC also blocked TGF-mediated activation of both EGFR and AKT. Overexpression of AKT by transient transfection substantially blocked the apoptosis inducing effects of PEITC. Our results showed that PEITC inhibits ovarian tumor growth *in vivo* by suppressing EGFR-AKT pathway and identifies EGFR-Akt axis as a target of PEITC.

Constitutive activation of EGFR has been reported in various cancers including breast, prostate and ovarian [33]–[35]. EGFR family of proteins is vital for cell growth. Activated EGFR is diversely involved in transducing growth signal and inhibiting apoptosis. In addition, it is also involved in angiogenesis, EMT and metastasis [9], [10]. A study conducted by Alper and colleagues have shown that approximately 70% of ovarian tumors express high levels of EGFR [36]. Inhibition of EGFR activation has been shown to suppress the growth of human cancer cells [37], [38]. Moreover, most of the cancers acquire drug resistance due to the activation of EGFR pathway [3], [39]. Therefore, inhibiting EGFR could be one potential approach to treat ovarian cancer. Our results showed that PEITC treatment substantially suppressed the phosphorylation of EGFR at Tyr-1068 as well as constitutive protein expression of EGFR in different ovarian cancer cell lines. In agreement with these results, tumors from PEITC treated mice also showed drastic suppression of both active and constitutive expression of EGFR. Our results indicate that PEITC suppress the growth of ovarian cancer cells by disrupting the phosphorylation of EGFR. These results are in agreement with several studies which showed that chemopreventive agents such as diindolylmethane, resveratrol, capsaicin and silibilin suppress the growth of prostate, breast and lung cancer cells by targeting EGFR [40]–[43]. Studies by Trachootham et al., and Satyan et al. demonstrated the anti-cancer effects of PEITC in ovarian cancer cells through ROS generation and MAPK activation [44], [45]. Our current study indicates an additional mechanism of action of PEITC in ovarian cancer cells.

AKT is pivotal to EGFR activation. Activated EGFR further activates AKT by phosphorylating it at Ser 473 [46]. AKT is a potent survival pathway that may mediate resistance to the apoptosis inducing effects of chemotherapy and radiation therapy in a variety of cancer types including ovarian cancer [47]. Several studies have shown the involvement of AKT signaling in apoptosis [48]. AKT is frequently overexpressed in ovarian cancer and plays a major role in ovarian carcinogenesis [14], [15]. Overexpression of AKT is frequently associated with aggressive phenotype and poor prognosis of ovarian cancer. Blockade of AKT has been shown to cause apoptosis in breast and pancreatic cancers [16], [49], [50]. Our study reveals that PEITC blocks both the activation and protein expression of AKT in all the three ovarian cancer cells. PEITC mediated inhibition of AKT was further verified by kinase activity of AKT by determining the phosphorylation of GSK. Several studies demonstrated the association of AKT activity with EGFR activation [51], [52]. Our results also showed that PEITC treatment reduced mTOR, Raptor and Rictor indicating that PEITC targets mTORC1 and mTORC2 complexes. Curcumin and fisetin also were shown to modulate the expression of mTOR, Rictor and Raptor in colorectal and prostate cancer cells respectively [53], [54]. The immunoprecipitation studies demonstrated that the expression of mTOR-associated Rictor was reduced more than Raptor by PEITC treatment. These observations suggest that the mTORC2 complex was affected more by PEITC treatment than mTORC1 complex. Since mTORC2 complex regulate the activation of AKT in cancer cells, our results suggest that reduced phosphorylation of AKT by PEITC treatment was primarily associated with the disruption of mTORC2 complex. Our results are in agreement with the studies published by Toschi et al. demonstrating the dissociation of Rictor from mTORC2 complex to enhance cell death by Rapamycin [55]. Furthermore, exposure of cells to TGF, a ligand of EGFR substantially increased the activation (phosphorylation) of AKT but suppressed by PEITC. These observations indicate that EGFR is upstream and pivotal to the activation of AKT in our model. Our results also showed that ovarian tumor growth suppression by PEITC was associated with the inhibition of AKT phosphorylation and expression. Our results thus established AKT as a target of PEITC in ovarian cancer cells.

It is important to note that oral administration of PEITC has reasonable bioavailability. Oral administration of 10 µmol PEITC in rats resulted in approximately 9.2±0.6 µM PEITC in the plasma after 0.44 hour of treatment [56]. In another study performed in healthy human volunteers, consumption of 100 g watercress by human volunteers resulted in approximately 928±250 nM PEITC in plasma [57]. In yet another study, consumption of a single hydrolyzed extract of 3-day old broccoli sprouts (containing approximately 200 µM total isothiocyanate) resulted in peak concentration of 0.94 to 2.27 µM isothiocyanates in plasma and serum within 1 hour of broccoli consumption in humans [58]. These results indicate that the therapeutic concentration of PEITC can be achieved. It is important to note that PEITC is currently under clinical trials for lung cancer. Outcome of the clinical trial would further validate the use of PEITC as a therapeutic agent against various cancers including ovarian cancer. Several EGFR targeted therapies such as monoclonal antibodies, small molecule inhibitors or RTK inhibitors failed to pass phase II clinical trials of ovarian cancer [59], giving rationale to develop newer therapies.

In conclusion, our results established that PEITC suppresses the growth of ovarian cancer *in vitro* and *in vivo* by inhibiting EGFR signaling. Our results also provide evidence that PEITC suppress the phosphorylation of AKT, which is regulated by EGFR. Taken together, our study provides support for the use of PEITC in pre-clinical and clinical settings for the management of ovarian cancer.
